# The Development and Validation of a Cognitive Diversity Scale for Chinese Academic Research Teams

**DOI:** 10.3389/fpsyg.2021.687179

**Published:** 2021-12-07

**Authors:** Feng Dong, Jian Peng, Xiao Wang, Minhui Tang

**Affiliations:** ^1^School of Management, Jinan University, Guangzhou, China; ^2^School of Management, Guangzhou University, Guangzhou, China

**Keywords:** team cognitive diversity, academic research team, scale development validation, Chinese, innovation

## Abstract

Cognitive diversity is an important concept stemming from western management research in the 1990s. With the rapid development of science and technology, there is a growing interest in the composition of an academic research team, such as team diversity. However, there is no tool available for measuring team cognitive diversity (TCD) for academic research teams. Based on Van der Vegt’s theoretical model of TCD, an Academic Research Team Cognitive Diversity Scale (ATCDS) is developed and validated for an academic research team in our research with two studies (N=737). In Study One, in-depth interviews and panel discussions were conducted to generate a preliminary questionnaire. In Study Two, the questionnaire was administered among academic research teams. Exploratory factor analysis revealed four factors regarding cognitive diversity: (1) the way of thinking, (2) knowledge and skills, (3) the view of the world, and (4) beliefs about what is right and wrong. The factor structure was further validated by confirmatory factor analysis. Moreover, correlation and regression analyses showed that academic research TCD was positively related to team creativity (*r* =0.306, *p* <0.01) and performance (*r* =0.204, *p* <0.10). To sum up, our newly developed 15-item ATCDS is sufficiently reliable and valid to be used for understanding cognitive diversity among academic research teams.

## Introduction

The work structure in academic environments has become increasingly team-based, particularly in the context of the research project, in which academic staff and researchers tend to work collaboratively within a team (Henkel, 2007). An academic research team is defined as a group of researchers who have complementary skills, are willing to take joint responsibility for common scientific research goals, and cooperate to produce scientific results through formal or informal collaboration (Cohen, 1991). Diversity among team members is an essential factor influencing team performance and can produce both beneficial and detrimental effects on team functioning (Harrison and Klein, 2007). The cognitive resource diversity theory is the most acceptable theory to explain the positive function of diversity and its relationship with performance. The cognitive resource diversity theory states that diversity has a positive impact on the team’s performance because of the unique combination of cognitive resources that members bring to the team (Hambrick et al., 1996). Among the different types of team diversity, cognitive diversity is particularly relevant to the outcomes of the academic research team (Harrison et al., 2002). When team members differ in terms of knowledge or skills, a team with cognitive diversity will emerge. Cognitive diversity facilitates the exchange of information and feedback, thus helping explain the positive effect of diversity on the team’s performance (Hambrick et al., 1996). Notably, cognitive diversity is the most relevant to analyze its effects on academic research teams, as it provides different perspectives, ideas, and thoughts that are required to generate creative ideas and facilitate creative processes.

Generally, “team cognitive diversity” refers to the extent to which the team members differed in their way of thinking, in their knowledge and skills, values, and beliefs (Van der Vegt and Janssen, 2003; Dahlin et al., 2005; Shin et al., 2012). Although research on cognitive diversity is increasing, previous studies have yielded contradictory results with regard to the effect of cognitive diversity on team performance (Bell et al., 2011; van Dijk et al., 2012). One important reason for the mixed results is the lack of a suitable, precise, and operable measure for cognitive diversity. The measurement of cognitive diversity mainly includes proxy variable approach and questionnaire-based approach. First, the proxy variable method directly measures cognitive diversity with surface-level (demographic) diversity, such as educational diversity, functional diversity, and work experience diversity (Miller et al., 1998). This method is somewhat more intuitive and easier to measure. The disadvantage is that it is not comprehensive enough, because it is challenging to measure deep-level cognition with one or two proxy variables, and it can only be said that these variables are partly related to cognition. Second, the questionnaire method indirectly measures deep-level (psychological) cognitive diversity through scales (Van der Vegt and Janssen, 2003). The questionnaire method can better measure and capture the deep-level diversity, such as cognitive diversity. Although some studies developed items to measure cognitive diversity, they failed to follow the rigorous procedures of psychometrics (Van der Vegt and Janssen, 2003). Nor do we know about the structure and scales of cognitive diversity. That is, there still lacks a reliable scale to measure the cognitive diversity of academic research teams. To the best of our knowledge, no cognitive diversity scale has been developed for the academic research team.

Although the concept of Van der Vegt and Janssen’s (2003) team cognitive diversity (TCD) and his four-item measurement are generally accepted, the scale is mostly applied to workgroups among firms. It has been noted that the characteristics of an academic research team are significantly different from those of a work team in the firm as an academic research team’s focus is more on knowledge development, while a work team in the firm emphasizes the application of knowledge (Alvesson, 2004). Accordingly, neither the measurement construct nor the content of TCD will be different when research is conducted in an academic research team. Therefore, it is necessary to develop a new scale to match the context of academic work to enhance the measurement validity (Hinkin, 1995).

The primary purpose of this study was to develop an Academic Research Team Cognitive Diversity Scale (ATCDS) and to examine its psychodynamic properties in Chinese academic research teams. Based on the scale development procedures of Hinkin (1995), we developed a more comprehensive cognitive diversity scale that is suitable for the Chinese context with regard for commonly accepted international standards.

## Study 1: Scale Development

Development of the ATCDS proceeded in the following sequential steps. Step 1, in-depth interviews with research team leaders or team members were conducted to generate a list of potential items for measuring academic research TCD. Step 2, panel discussions were conducted to rationalize and reduce the number of items to construct a preliminary questionnaire.

### Step 1: Generation of Potential Items

According to the definition of cognitive diversity (Van der Vegt and Janssen, 2003), the participants were required to fully describe the characteristics of team members based on their experience and observations. The participants’ criteria include as: (1) Participants should be researchers of academic research teams in Chinese Universities or research institutes; (2) Participants should work in the team for more than 1year and be familiar with the team function and team members; and (3) The team should consist of a leader and more than two core members (given that a team should have more than three persons). Moreover, we consider the team size, team academic background, member age, tenure, and title, on which we base to choose the representative teams whose members are diverse in gender, age, title, functions, and academic backgrounds. Research team leaders (*n*=4) and members (*n*=4) were invited to participate in an in-depth interview to identify sources of cognitive diversity within an academic research team. The number of participants was determined based on the saturation method (Saumure and Given, 2008), where data were collected to the point where subsequent participants fail to provide unique information on the topic under investigation. When we interviewed the eighth participant, the information he/she provided is similar to those provided by the former seven participants, without unique information. Then, we decided to finish the interview. The eight participants were recruited from three universities in Guangzhou, China, representing a range of ages, genders, and research backgrounds. The average tenure of the team members was 8years. All participants were interviewed individually and each interview lasted between 60 and 90min. The interviews were conducted in a quiet room by three trained research assistants. The examples of interview questions were as: “Do you think there are differences among team members, and what are the specific differences?,” “Are there differences among members in terms of attitudes, viewpoints, ways of solving problems and thinking styles of new knowledge, new methods, and new technologies in the process of jointly completing scientific research projects?” The interviews were tape-recorded and were converted into a document of 134,500 words by the NVIVO 11 software (QSR international company, Australia). The document was interpreted by qualitative research methods (Malterud, 2001). By manually encoding and extracting items, 156 descriptive items were initially generated.

### Step 2: Item Screening

First, we deleted the repetitive descriptive items and non-cognitive items, and excluded descriptions about behaviors and attitudes. For example, “The differences over accepting new perspectives among team members” and “Team members are willing to try new research perspectives.” This step resolved to 83 descriptive items. Second, the items with similar descriptions were combined. For instance, the differences in knowledge and skills of team members were combined as “diversity of knowledge and skills”; the differences of thinking styles among team members were combined as “diversity of thinking styles.” This step resulted in a list of 43 items. There are three experts (and seven Ph.D. candidates) taking part in the item screening stage. Following Hinkin’s (1998) recommendation, we conducted an item screening study to examine the content validity of the ATCDS. We asked three experts and seven Ph.D. candidates (in the research field of organizational behavior) to match items into three specific categories (i.e., cognitive diversity, demographic diversity, and other categories). The results showed that 23 items were assigned to the cognitive diversity category more than 80% of the time. Thus, 23 items were retained.

## Study 2: Validation of the Scale

### Samples

Three samples (Sample One, Sample Two, and Sample Three) were obtained for the conduct of exploratory factor analysis (EFA), confirmatory factor analysis (CFA), and criterion reliability, respectively. The scale was distributed to academic research teams in universities or research institutions in Beijing, Shanghai, Guangdong, Jiangsu, and Liaoning provinces in China. The employed sample conforms to the definition of the academic research team. For Sample One, from June to July, 2020, a total of 308 questionnaires were distributed by email and 299 questionnaires were returned (93.43%). The respondents were from a wide array of universities in China. A total of 58.25% had a professor title, 28.28% had an associate profession title, and 10.77% had an assistant professor title. In addition, 4.35% had a national-level talent title, 18.73% had a province-level talent title, and 76.92% did not have a talent title. Moreover, regarding the research fields, 11.04% were Engineering Science, 17.21% were Science, 10.06% were Medical Science, 41.23% were Life Science, and 20.45% were Social Science. Moreover, 165 were male (55.18%) with a mean age of 37years (SD=6.47), and 233 were team members (77.92%) and the remaining were team leaders.

For Sample Two, from July to April, 2020, a total of 362 questionnaires were distributed by email and 308 questionnaires were returned (85.10%). The respondents were from a wide array of universities in China. A total of 52.60% had a professor title, 19.48% had an associate profession title, and 14.61% had an assistant professor title. In addition, 6.17% had a national-level talent title, 8.44% had a province-level talent title, and 85.39% did not have a talent title. Moreover, regarding the research fields, 29.22% were Engineering Science, 15.58% were Science, 25.00% were Medical Science, 24.35% were Life Science, and 5.84% were Social Science. Moreover, 180 were male (58.40%) with a mean age of 33.63years (SD=8.05), and 262 were team members (85.06%) and the remaining were team leaders.

For Sample Three, from September to October, 2020, a total of 150 questionnaires were distributed by email and 130 questionnaires were returned (86.67%). The respondents were from a wide array of universities in China. A total of 70.77% had a professor title, 11.54% had an associate profession title, and 3.08% had an assistant professor title. A total of 24.62% had a national-level talent title, 27.69% had a province-level talent title, and 47.69% did not have a talent title. Moreover, regarding the research fields, 27.69% were Engineering Science, 13.08% were Science, 25.38% were Medical Science, 28.46% were Life Science, and 5.38% were Social Science. A total of 102 were male (78.46%) with a mean age of 41.96years (SD=10.23).

All procedures performed in studies involving human participants were in accordance with the ethical standards of Jinan University. The approval document is available upon request.

### Item Analysis

Item analysis was performed to calculate the item means, standard deviations, and item-to-total correlations. The results showed that the item-to-total correlations range from 0.302 to 0.688. No item was removed because the item-total correlation coefficients were above the threshold criteria of 0.30 (Nunnally and Bernstein, 1994). A t-test was performed to test the critical ratios (composition reliability; CR) of each item. All of the items that exhibited CR ranging between 5.07 and 17.05, which met the set criteria (CR>4), were retained and were all statistically significant (*p*<0.001). Meanwhile, a Cronbach’s α of 0.92 was the threshold level of homogeneity. As the alpha coefficient for item 9 exceeded 0.92, item 9 was deleted from further consideration.

### Exploratory Factor Analysis

Using Sample One, we performed a principal components analysis (varimax rotation) on the pool of 15 items to examine whether four meaningful factors representing “Way of thinking,” “Knowledge and skills,” “Beliefs about right and wrong,” and “World view” could be obtained. We aimed to develop a reliable instrument while avoiding an overly exhaustive scale containing too many items to be used conveniently. So, while we deliberately started with a relatively large pool of items to empirically answer the question of which items functioned best together in terms of their loadings, one of our goals was to reduce the number of items significantly.

A series of statistics indicated that EFA is appropriate for the current dataset: Kaiser–Meyer–Olkin (KMO) =0.89, Bartlett’s *p*<0.001. The EFA results suggested extracting four factors, which account for 68.08% of the total variance. The four factors were defined, respectively, including “way of thinking” (five items), “knowledge and skill” (three items), “beliefs about what is right or wrong” (four items), and “world view” (three items). However, 7 of the 22 items, including item 6, item 16, item 19, item 20, item 21, item 22, and item 23, had weak factor loadings (<0.50) or high cross-loadings, suggesting the removal of these items (Tabachnick and Fidell, 2013). Internal consistency reliability of the 15-item scale was evaluated by Cronbach’s alpha and inter-item correlation. The results yielded a Cronbach’s alpha of 0.87, indicating good internal consistency (DeVellis, 2003). The average inter-item correlation for all items was 0.30, which was acceptable and suggested that the items measure the same construct well.

### Confirmatory Factor Analysis

Using Sample two, we performed CFA on the 15 items using AMOS software (Arbuckle, 2013). To assess model fit, six different fit indices were used. For absolute model fit, *χ*^2^/df, the goodness-of-fit index (GFI), the root mean square residual (RMR), and the root mean square error of approximation (RMSEA) were examined. In addition, for relative model fit, we examined the Normed Fit Index (NFI) and the comparative fit index (CFI). Values of 0.08 and under (for RMR and RMSEA) or 0.90 and over (for CFI, NFI, and GFI) indicate acceptable fit (Hu and Bentler, 1999; Byrne, 2001). Thus, the results generally indicated an acceptable fit for the four-factor model.

**Figure 1:**
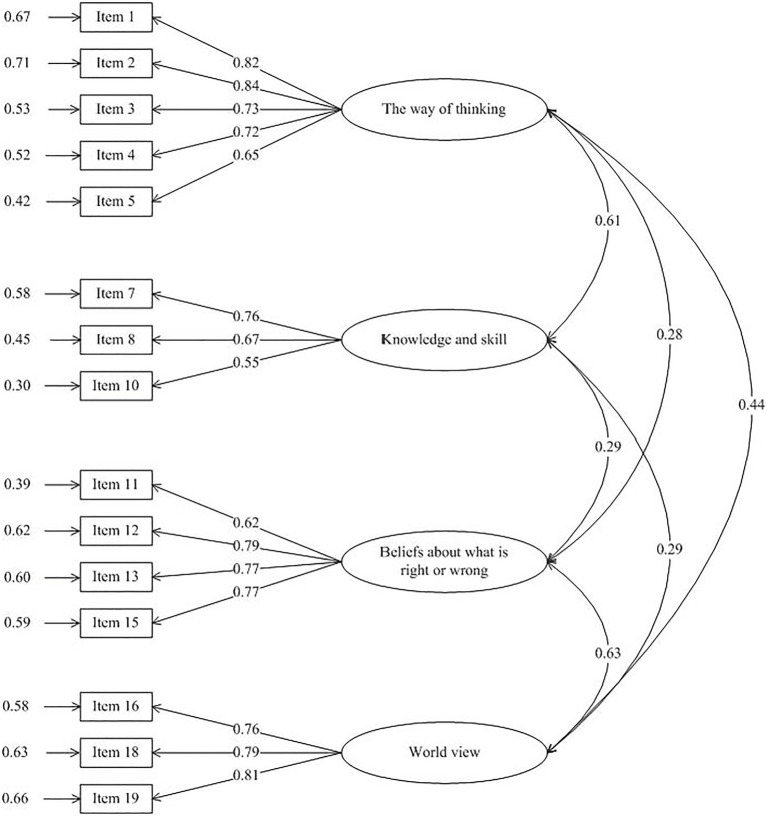
Estimations of standardized path coefficient of the final confirmatory factor model.

The model derived by EFA was then validated by CFA. The results demonstrated that our model had excellent fit with the data (CFA: *χ*^2^/df=2.37, NFI =0.90, CFI =0.94, GFI =0.91, RMSEA =0.07, RMR =0.05). The graphical expression of the path diagram of the revised EFA model is presented in Figure 1. The factor loadings for each item ranged from 0.55 to 0.84.

### Criterion Validity

#### Creativity and Performance

We verified the 15-item Academic Research Team Cognitive Diversity Scale (ATDCS) validity using team creativity and performance as the criterion (Miller et al., 1998; Shin et al., 2012). Cognitive diversity reflects the difference in the beliefs, thinking styles, knowledge, values, assumptions, and preferences held by the team’s members. The greater the cognitive diversity of a team, the easier it is to get the different cognitive resources, which means get different perspectives, ideas, and styles of thought. Cognitive diversity provides the academic research team with a pool of knowledge resources that may help to deal with scientific problems, which requires a more creative view. Therefore, cognitive diversity influences the resources of creativity, all of which have a positive effect on the team’s creativity (Kanchanabha and Badir, 2021). It shows that team deep-level diversity is associated with fewer positive emergent states and positive team processes and more team conflict. There is an indirect relationship between TCD and team performance through each of the mediators: absorptive capacity (Schweisfurth and Raasch, 2018; Triana et al., 2021). Based on these considerations and arguments, the following hypothesis is proposed, respectively:

*Hypothesis 1: Cognitive diversity in academic research teams is positively related to team creativity*.

*Hypothesis 2: Cognitive diversity in academic research teams is positively related to team performance*.

#### Results of Criterion Validity

Creativity and performance were measured with two adapted scales from Huang (2016) research on original innovation among universities. In this study, each participant completed a questionnaire of three scales and reported their demographics. Team cognitive diversity was measured with the newly developed ATCDS (Table 1). Cronbach’s α was. 865. Five-point Likert scales were used with answers ranging from 1 (strongly disagree) to 5 (strongly agree). We controlled for gender, age, team tenure, and team size.

**Table 1:** Exploratory factor analysis of final 15 items (structure of ATCDS; N=299).

Items	Factors				Way of thinking	Knowledge and skills	Beliefs about right and wrong	World view
*The extent to which the team members differ in*				
Refining scientific question	0.79			
Problem-thinking approaches	0.81			
Problem-solving approaches	0.77			
Decision making	0.82			
Task-influencing factors	0.69			
Educational specialization		0.75		
Professional skills		0.81		
Educational level		0.68		
Belief that members have different Functions			0.75	
Belief that researchers should be on the front-line			0.81	
Belief that adhering to rigorous academic norms and ethics			0.79	
Belief that research tasks need to be outsourced			0.74	
Religious beliefs				0.86
Political beliefs				0.81
The origin of the world				0.74

*Factors were extracted using principal component factor analysis. The rotation was processed using the varimax method*.

We conducted all analyses in this part using Stata 15.0. Table 2 shows the descriptive statistics for all variables. Most academic research team leaders in this study were males (78%); the average age of academic research team leaders was 41.96years; and all of the teams recorded high creativity (mean=3.92, of a maximum score of 5).

**Table 2:** Descriptive statistics of team cognitive diversity and criterion variables.

	Min	Max	Mean	SD
Gender	0	1	0.78	0.41
Age(*years*)	23	64	41.96	10.57
Team tenure(*years*)	2	23	7.67	4.83
Team size(*N*)	2	60	15.12	11.91
Team cognitive diversity	2	5	3.48	0.45
Creativity	2	5	3.92	0.60
Performance	2	5	3.58	0.62

Table 3 shows the correlations for all variables.

**Table 3:** Correlations of team cognitive diversity and criterion variables.

	1	2	3	4	5	6	7
1. Gender	1						
2. Age	0.10	1					
3. Team tenure	−0.17^*^	0.36^***^	1				
4. Team size	0.05	0.06	0.28^***^	1			
5. Team cognitive diversity	−0.06	−0.08	0.05	0.09	1		
6. Creativity	0.16+	0.22^**^	−0.01	0.25^**^	0.22^**^	1	
7. Performance	0.08	0.04	0.13	0.40^***^	0.16+	0.48^***^	1

N=130, + p<0.10, ^*^p<0.05, ^**^p<0.01, and ^***^p<0.001.

To test our hypothesis that TCD has positive impacts on creativity and performance, we constructed four models. Models 1 and 3 only contained the control variables, while Models 2 and 4 added the independent variable, TCD. The results of the four models in Table 4 show that TCD has a positive impact on team creativity (*r*=0.306, *p*<0.01) and team performance (*r*=0.204, *p*<0.10). Meanwhile, compared to Model 1, the R square of Model 2 increased by 5%. Also R square of Model 4 increased by 2% contrasted to Model 3. The increasing R square of Models 2 and 4 proved the effects of TCD on creativity and performance. In general, the above analyses provide criterion validity for our ATCDS.

**Table 4:** Regression results.

	Creativity		Performance	
	Model 1	Model 2	Model 3	Model 4
Constant	3.58^***^	2.53^***^	2.92^***^	2.22^***^
Control variables				
Gender	0.16	0.19	0.21	0.23^*^
Age	0.02	0.02	−0.00	−0.00
Team tenure	−0.01	−0.01	0.01	0.01
Team size	0.09	0.07	0.29^***^	0.28^***^
Independent variables				
Cognitive diversity		0.31^***^		0.20+
R^2^	0.30	0.35	0.25	0.27

N=130, + p<0.10, ^*^p<0.05 and ^***^p<0.001.

## Discussion

In developing a scale of ATCDS while extending the prior scale of CD influence on work group in firms, we make three contributions to CDS. First, we offer a new, robust, and valid tool to measure CD in the academic research team. The proposed 15-item scale offers adequate psychometric properties, as indicated by strong, consistent evidence across a pilot study and a main study with distinct samples of team leaders or members (N=737). By using multiple, independent, relatively large samples from a broad spectrum of settings, we improve the generalizability of our findings while also accounting for the specific contexts of CD. We find strong support for the psychometric properties of ATCDS, in terms of content and criterion-related validity. The emerging stream of studies on the CD and performance can benefit from this new ATCDS and test its theoretical predictions. Second, we provide an academic research team conceptualization of CD with four subordinate dimensions. That’s “way of thinking,” “knowledge and skills,” “beliefs about right and wrong” and “world view.” Prior scale developments have not always followed the standards to demonstrate multidimensional construct validity and evidence. We show that ATCDS is an overall, superordinate, multidimensional construct organized.

Our ATCDS differs from the past cognitive diversity scale in work teams in two important ways. First, it focuses on an essential yet different context, namely, the academic research team in China. Academic research teams focus more on knowledge development, while work teams in corporates emphasize the application of knowledge. Second, the development and validation of ATCDS strictly follow the rigorous process of scale development by Item Generation, Scale Development, and Scale Evaluation (Hinkin, 1995). Following the existing literature that the measures should demonstrate content validity, internal consistency, construct validity, and criterion validity, this study performed several tests, and the final 15-item ATCDS shows excellent reliability and validity by psychometric principles.

At first, eight participants were interviewed, and a document of 134,500 words was formed. We manually encoded and extracted 156 descriptive items. After that, a panel of three experts analyzed a list of 43 items, to ensure that each item is reliable and without repetition, so as to guarantee a good content validity. Then, comparing to the common cognitive diversity scale of Van der Vegt and Janssen (2003) with a Cronbach’s alpha of 0.81 in work teams, the Cronbach’s alpha of our ATCDS reached 0.87, indicating good reliability when used in the academic research team. Meanwhile, the result of a principal components analysis with varimax rotation method on the pool of 15 items shows that four significant factors representing “Way of thinking,” “Knowledge and skills,” “Beliefs about right and wrong,” and “World view” could be obtained. The model was also validated by a CFA. The results demonstrated that our model had excellent fit with the data (CFA: *χ*^2^/df=2.37, NFI =0.90, CFI =0.94, GFI =0.91, RMSEA =0.07, RMR =0.05). Finally, the results of the regression analysis showed that cognitive diversity using our ATCDS has a positive impact on team creativity (*r* =0.306, *p* <0.01) and team performance (*r* =0.204, *p* <0.10), indicating the ATCDS has a good criterion validity. In sum, the analyses used for reliability and validation support the development of our ATDCS.

To our best knowledge, this is the first study to develop a scale assessing cognitive diversity in the Chinese academic research team and validate its psychometric properties. We offer a new, robust, and valid tool to measure perceptions of cognitive diversity in the academic research team, which serves as a complementary to Van der Vegt and Janssen’s (2003) scale. Van der Vegt and Janssen (2003) have some shortages, such as questionable reliability and validity and limited generalizability in academic research teams. To address this issue, we draw upon the definition of cognitive diversity and followed the rigorous process of scale development (i.e., Item Generation, EFA, CFA, and test of reliability and validity) to develop ATCDS. This scale has many strengths. First, ATCDS has higher reliability and validity than Van der Vegt and Janssen (2003). Compare with Van der Vegt and Janssen’s (2003) four-item scale, our ATCDS has 15 items (loading on four factors), which can capture and measure cognitive diversity more comprehensively, accurately, and reliably. Second, ATCDS focuses on an important yet different context, namely, the academic research team in China. Notably, the characteristics of academic research teams are significantly different from those of a work team in corporates such that academic research teams focus more on knowledge development, while work teams in corporates emphasizes the application of knowledge.

To provide a more accurate measurement for TCD in academic work, we conduct two studies to develop and test a new scale with four dimensions. The data were collected from a review of previous studies, in-depth interviews with eight interviewees, 299 questionnaires for EFA, and 308 questionnaires for CFA. Finally, this research developed a four-dimension 15-item ATCDS for Chinese academic research teams. Our work provides several contributions to TCD research.

First, we explore the conceptual richness of cognitive diversity in academic work. Although the four-item single-dimension scale of Van der Vegt and Janssen (2003) has been widely used, it fails to reflect the comprehensive nature of cognitive diversity accurately (Mitchell et al., 2016). Our four-dimension, 15-item scale authentically and explicitly shows the cognitive diversity among academic research teams in China. In general, individuals who reason at a higher cognitive developmental level tend to use a broader range of thinking styles than individuals who reason at a lower cognitive developmental level (Hamid, 2017). Because the thinking style, knowledge, skill, belief, and worldview can impact the cognitive process of team members (Shin et al., 2012), all four dimensions are vital to constitute the ATCDS. Based on the previous four items, the corresponding four-dimensional structure is obtained, and our results prove that the ATCDS has high reliability and validity, enriching the measurement of TCD.

Second, the four dimensions we proposed for our new scale provide a more comprehensive understanding of academic work. For example, with regard to the dimension of thinking styles, it is the kind of disposition in which people organize or ponder their responses and attitudes toward certain events or work (Kim and Song, 2013). It focuses on the process of how people respond to an event. For an academic research team, thinking styles are aspects that can impact team members’ responses or attitudes toward the research. Thus, the way team members refine a scientific question, thinking and solving-related problems, making decisions, and considering task-influencing factors are all significant elements of an academic research teams’ thinking style.

As to the knowledge and skill dimension of cognitive diversity in academic work, the cognitive underpinnings depend on the team’s understanding of a complex and dynamic situation at any one point in time (i.e., team situation awareness) and is supposedly influenced by the knowledge that the team possesses (Stout et al., 1996). Team knowledge consists of background knowledge that is long-lived in nature, as well as more dynamic, differing from the fleeting understanding that an operator may have of a situation at any one point in time (Cooke et al., 2001). Thus, the long-term knowledge of an academic research team is composed of the diversity of educational specialization, professional skills, and educational levels among team members. Although previous research has also focused on the single aspect of knowledge and skills, for instance, educational specialization (Shin and Zhou, 2007) and educational levels (Bell et al., 2011), our study combines diverse aspects of knowledge and skills as a whole.

Since the seminal work of Van der Vegt and Janssen (2003), beliefs about what is right and wrong have been an important content of TCD. Although research shows that team members’ beliefs are formed through constant interaction and communication among team members and a collective sense-making process (Boyce, 1995), each team member’s belief originates from individual motivation and could be diverse. We argue that researchers in academic teams should be on the front-line, adhering to rigorous academic norms and ethics, and research tasks need to be outsourced.

Finally, as to the worldview dimension of cognitive diversity in academic work, it is a concept of attitude rather than knowledge (McGaha and Linder, 2014). Although knowledge is important, it is the attitudes that “influence which knowledge is noticed, sought, and developed” (Parker et al., 1997). The attitude is shaped by the individual’s interaction with their environment. Worldviews emerge from a cultural milieu including religion, politics, science, place-based values, education, and ethnicity (Curry, 2000). Thus, the fundamental questions, such as religious beliefs, political beliefs, and the origin of the world, can largely reflect an individual’s worldview.

## Limitations and Directions For Future Studies

The current research has some limitations. First, the geographical regions within which the academic research teams were sampled were limited. Although we attempted to obtain typical and representative information and included both developed and undeveloped geographical areas, certain provinces or municipalities were not included in this research. To improve the generalization of ATCDS, we encourage future research to collect new samples from various regions to retest the validity of ATCDS. Second, the objective of this research was to develop an ATCDS for the Chinese academic research team, whose culture emphasizes collectivism that people view themselves as interdependent entities and maintain social harmony, hierarchy, and obedience (Hofstede, 1980). That said, whether this scale can be applied to other countries remained under-explored. We encourage future research to examine the reliability and validity of ATCDS in western countries with individualistic cultures where people view themselves as bounded entities and pursue their individual autonomy and uniqueness (Hofstede, 1980). Third, the current research only considered two criteria (creativity and performance) when testing criterion validity, we encourage future research to examine the predictive value of ATDCS further. As Kong and Jolly (2019) posited, research should provide more information about the predictive value of a new scale. In this vein, future research could explore what other team outcomes ATDCS can predict. We expect that more effort can be devoted to exploring both in-role and extra-role consequences of ATDCS. Finally, although we found cognitive diversity positively affected team creativity and performance, the mechanism underlying this relationship is not yet fully understood. Kim et al. (2021) suggested an indirect relationship between TCD and team performance through some mediators, such as motivation, information exchange, and absorption ability. Hence, a promising direction for future research may be to explore the mediators between cognitive diversity and team outcomes in academic research teams.

## Conclusion

This research used a qualitative analysis method to develop an initial ATCDS. Across multiple studies, this study tested the initial psychometric properties of the four-dimension-15-item ATCDS. The results of item analysis, EFA, CFA, and criterion validity show that the initial 15-item ATCDS has a decent reliability and validity. Therefore, it is helpful for scholars to further investigate cognitive diversity of the ATCDS measure among academic research teams.

## Data Availability Statement

The original contributions presented in the study are included in the article/supplementary material, and further inquiries can be directed to the corresponding author.

## Ethics Statement

The data were collected using an online-survey among Chinese research teams who were invited *via* email and gave their informed consent that researchers can analyze the collected data for scientific purposes. The common Ethics Commission of the Jinan University gave ethical approval for data collection. The patients/participants provided their written informed consent to participate in this study.

## Author Contributions

FD contributed to data collection, data analysis, discussion of results, and written and preparation of the manuscript. JP and XW contributed to the conception of the study and analysis with constructive discussions. MT contributed to study design, data collection, results interpretation, discussion of results, and written and preparation of the manuscript. All authors contributed to the article and approved the submitted version.

## Funding

We acknowledge the financial support from the National Natural Science Foundation of China (nos. 71922011 and 71772076).

## Conflict of Interest

The authors declare that the research was conducted in the absence of any commercial or financial relationships that could be construed as a potential conflict of interest.

## Publisher’s Note

All claims expressed in this article are solely those of the authors and do not necessarily represent those of their affiliated organizations, or those of the publisher, the editors and the reviewers. Any product that may be evaluated in this article, or claim that may be made by its manufacturer, is not guaranteed or endorsed by the publisher.
